# Reducing confounding and suppression effects in TCGA data: an integrated analysis of chemotherapy response in ovarian cancer

**DOI:** 10.1186/1471-2164-13-S6-S13

**Published:** 2012-10-26

**Authors:** Fang-Han Hsu, Erchin Serpedin, Tzu-Hung Hsiao, Alexander JR Bishop, Edward R Dougherty, Yidong Chen

**Affiliations:** 1 Department of Electrical and Computer Engineering, Texas A&M University, College Station, TX, USA; 2 Computational Biology Division, Translational Genomics Research Institute, Phoenix, AZ, USA; 3 Greehey Children's Cancer Research Institute, University of Texas Health Science Center at San Antonio, San Antonio, TX, USA; 4 Department of Cellular and Structural Biology, University of Texas Health Science Center at San Antonio, San Antonio, TX, USA; 5 Department of Epidemiology and Biostatistics, University of Texas Health Science Center at San Antonio, San Antonio, TX, USA

## Abstract

**Background:**

Despite initial response in adjuvant chemotherapy, ovarian cancer patients treated with the combination of paclitaxel and carboplatin frequently suffer from recurrence after few cycles of treatment, and the underlying mechanisms causing the chemoresistance remain unclear. Recently, The Cancer Genome Atlas (TCGA) research network concluded an ovarian cancer study and released the dataset to the public. The TCGA dataset possesses large sample size, comprehensive molecular profiles, and clinical outcome information; however, because of the unknown molecular subtypes in ovarian cancer and the great diversity of adjuvant treatments TCGA patients went through, studying chemotherapeutic response using the TCGA data is difficult. Additionally, factors such as sample batches, patient ages, and tumor stages further confound or suppress the identification of relevant genes, and thus the biological functions and disease mechanisms.

**Results:**

To address these issues, herein we propose an analysis procedure designed to reduce suppression effect by focusing on a specific chemotherapeutic treatment, and to remove confounding effects such as batch effect, patient's age, and tumor stages. The proposed procedure starts with a batch effect adjustment, followed by a rigorous sample selection process. Then, the gene expression, copy number, and methylation profiles from the TCGA ovarian cancer dataset are analyzed using a semi-supervised clustering method combined with a novel scoring function. As a result, two molecular classifications, one with poor copy number profiles and one with poor methylation profiles, enriched with unfavorable scores are identified. Compared with the samples enriched with favorable scores, these two classifications exhibit poor progression-free survival (PFS) and might be associated with poor chemotherapy response specifically to the combination of paclitaxel and carboplatin. Significant genes and biological processes are detected subsequently using classical statistical approaches and enrichment analysis.

**Conclusions:**

The proposed procedure for the reduction of confounding and suppression effects and the semi-supervised clustering method are essential steps to identify genes associated with the chemotherapeutic response.

## Background

Ovarian cancer is prevalent in women [1] and is associated with a high mortality rate as it is usually diagnosed at an advanced stage [2]. A standard treatment of advanced ovarian cancer involves surgical resection followed by cycles of adjuvant chemotherapy, typically a combination of taxane-based regimens and platinum-based cytotoxic agents [3]. The combination of paclitaxel and carboplatin is one of the most common first-line treatments of ovarian cancer [4,5]. The mechanism of action (MOA) of paclitaxel is to stabilize microtubules and as a result it induces mitotic arrest and apoptosis [6], and the MOA of carboplatin is to bind with DNA and form intra-strand crosslinks so as to inhibit DNA replication and transcription, and eventually activate the p53-dependent apoptosis [7]. In most patients, the initial responses to the combination of paclitaxel and carboplatin are good; however, subsequent relapses frequently occur [8]. Unraveling the underlying mechanisms causing chemoresistance is crucial for personalized therapy and the improvement of patients' long-term survival.

Microarrays have been used to study genes and molecular functions associated with chemoresistance. For example, Jazaeri *et al. *(2005) detected differentially expressed genes among primary chemosensitive, primary chemoresistant, and postchemotherapy tumors using cDNA-based microarrays [9]. Additionally, Hartmann *et al. *(2005) applied a supervised learning algorithm and selected 14 genes to predict the relapsed outcome of ovarian cancer patients after platinum-paclitaxel chemotherapy [10]. Etemadmoghadam *et al. *(2009) further considered chromosomal aberrations and proposed that DNA copy number alterations (CNAs) at genes such as CCNE1 and NCOA3 are associated with chemoresistance [11]. While many studies had proposed genes or pathways associated with chemotherapeutic response, most of these studies suffered from limited number of patients and patient diversity, as well as other confounding factors to a certain extent, particularly when the samples were derived from patients with different treatment plans. Since these factors such as tumor stage, subtype, and different chemotherapies may change clinical outcome significantly, reliable results could be difficult to achieve if these confounding effects were not adequately addressed during statistical analysis.

In this regard, the Cancer Genome Atlas (TCGA) data need to be carefully assessed for eligibility to a chemotherapy study. Recently, the TCGA Research Network concluded an ovarian cancer study with thousands of microarray data including mRNA expression, DNA copy number, miRNA, SNP, and CpG methylation data from more than 500 ovarian tumor samples [12]. While a large number of samples provide ample opportunities to carry out sophisticated survival analysis, caution should be taken: patient ages, tumor stages and treatment cycles may confound the survival outcome, while various therapeutic compounds, their combination and sample processing batches may suppress the detection without proper handling. As an example, among more than 500 patients, treatments include avastin, bevacizumab, carboplatin, cisplatin, cytoxan, docetaxel, doxoribicin, etoposide, gemcitabine, navelbine, paclitaxel, and others. In addition, these samples were processed in 13 batches.

Herein, a procedure for reducing the confounding and suppression effects is proposed, in which, factors such as experimental batches, clinical treatment, patient ages, tumor stages, and molecular classifications are carefully considered and dealt with. Beginning with a batch effect correction, we chose eligible samples through a rigorous sample selection process. In this paper, we will focus only on patients with paclitaxel and carboplatin treatment in order to remove possible confounding factors due to better drug or treatment combination when examining the survival outcome, and in the meantime, to maximize the ability of discriminating tumor subtypes. After the selection, 85 ovarian cancer samples treated only with the combination of paclitaxel and carboplatin were selected for training, and another independent 83 samples treated mainly with the combination of paclitaxel and carboplatin but including some other drugs were applied for testing. Then, gene expression, copy number, and methylation data were analyzed in a novel semi-supervised clustering method. By performing a series of statistical hypothesis testing and clustering tasks, two molecular classifications with poor progression-free survival (PFS) were identified. Comparing these classifications to other samples with good PFS, genes significantly associated with chemotherapeutic response were detected, and enriched biological processes were further examined using a gene ontology enrichment analysis method.

In this paper, the proposed procedure and the semi-supervised clustering method are detailed with flow-charts and mathematical explanations in Methods. In Results, the clustering results and the subsequent differences in chemotherapeutic response are compared via Kaplan-Meier curves. Discussions of analysis results and conclusions are provided in Discussion and conclusions.

## Methods

Getting started from collecting data, this section demonstrates the methods and criteria we applied for achieving the results. First of all, we downloaded the ovarian cancer data from the TCGA repository website (http://cancergenome.nih.gov/). Level 3 gene expression data derived from Aymetrix U133A platform, level 2 copy number data derived from Agilent CGH-1x1M platform, and level 3 methylation data derived from Illumina HumanMethylation27 platform were chosen and downloaded. The gene expression data are constituted of 12,042 normalized log_2 _values, and each value represents an expression level of a gene. Copy number data contain 962,434 normalized log_2 _ratios among which 358,119 ratios with gene annotations were utilized. To match these copy number ratios with gene expression levels, values corresponding to a same gene were averaged. Methylation data are 27,578 beta values, and each value refers to the percentage of methylation for a specific CpG site. Among the 27,578 beta values, 19,448 linked to 10,068 unique genes included in the gene expression data were considered in this study. In total, a cohort of 514 tumor samples comprehensively containing gene expression, copy number, and methylation profiles was obtained.

**Figure 1:**
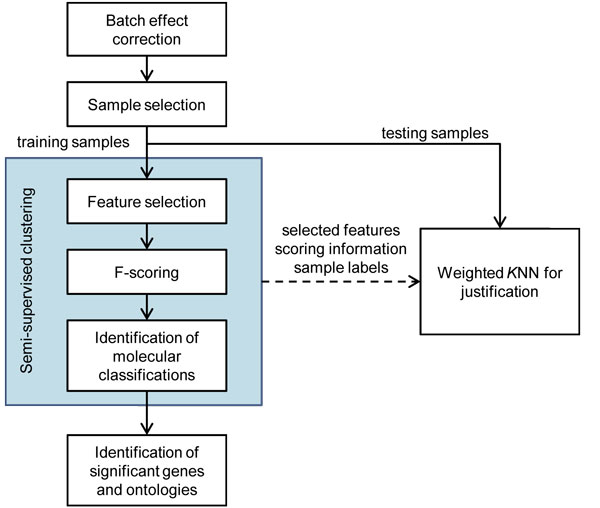
**The procedure**. The proposed procedure for the reduction of confounding effects and suppression effects.

The procedure for the reduction of confounding and suppression effects is described in the following subsections. As shown in Figure 1, the first step for using the TCGA data was batch effect correction. Then, a rigorous process for sample selection was proposed for finding two independent patient cohorts who went through the combination of paclitaxel and carboplatin treatment: one for training and one for testing. A semi-supervised clustering approach was further applied for identifying molecular classifications. Based on the identified classifications, further validated through testing patient cohort, differentially expressed genes were detected by comparing samples with good response to those with poor response, and significant ontologies were found using an enrichment analysis.

### Batch effect correction

Batch effect correction was applied to all 514 samples. Due to the large sample size, TCGA samples were derived from different institutions, and experiments were performed on different dates; these factors may cause quantitative differences in measurements and might lead to false discovery specifically if they are not properly dealt with [13]. In this study, location and scale (L/S) adjustments [14] were applied to eliminate batch effects in gene expression and methylation data. We did not perform batch correction to aCGH arrays due to the fact that it assumes a comparative hybridization to normal DNA on the same array; hence, it is less prone to batch effects. For gene expression, we assumed that the medians and variances of measures from different batches should be ideally the same. Let *g_i _*represents the vector of gene expression values for gene *i *from all 514 samples, *g_ij _*denotes the vector of gene expression values for gene *i *from samples corresponding to batch *j*, and *g_ijk _*represents the gene expression value for gene *i *from batch *j *and sample *k*. The adjusted gene expression value $g_{ijk}^{*}$ for gene *i *from batch *j *and sample *k *is given by

(1) $$g_{ijk}^{*}=M_{i}+\left(g_{ijk}-M_{ij}\right)\frac{{\hat{\sigma }}_{g_{i}}}{{\hat{\sigma }}_{g_{ij}}},$$

where *M_i _*refers to the median of *g_i_*, and *M_ij _*denotes the median of *g_ij_*. Also, ${\hat{\sigma }}_{g_{i}}$ and ${\hat{\sigma }}_{g_{ij}}$ are the estimated standard deviations of *g_i _*and *g_ij _*, respectively, and whose expressions are given by

(2) $${\hat{\sigma }}_{g_{i}}={\left[\frac{1}{N-1}\overset{N}{\underset{k=1}{\sum }}{\left(g_{ijk}-\bar{g_{i}}\right)}^{2}\right]}^{\frac{1}{2}},$$

(3) $${\hat{\sigma }}_{g_{ij}}={\left[\frac{1}{N_{j}-1}\underset{k\in K_{j}}{\sum }{\left(g_{ijk}-\bar{g_{ij}}\right)}^{2}\right]}^{\frac{1}{2}},$$

where *N *refers to the total number of samples, *N_j _*denotes the number of samples in batch *j*, and *K_j _*stands for the samples in batch *j*. The adjustment of methylation data is somewhat different since beta values have a bounded range (from 0 to 1) and exhibit a slight bimodal distribution [15]. Again, let *b_i _*and *b_ij _*represent two vectors of beta values for gene *i *across all 514 samples and batch *j*, respectively. To enforce beta values from different batches to have the same median, the methylation beta value *b_ijk _*for gene *i *from batch *j *and sample *k *is rescaled, and the adjusted value $b_{ijk}^{*}$ is given by

(4) $$b_{ijk}^{*}=\left\{\begin{array}{cc} b_{ijk}\frac{M_{i}^{\prime }}{M_{ij}^{\prime }}, & \;\text{if}\;b_{ijk}\leq M_{ij}^{\prime },\\ M_{i}^{\prime }+\left(b_{ijk}-M_{ij}^{\prime }\right)\frac{\left(1-M_{i}^{\prime }\right)}{\left(1-M_{ij}^{\prime }\right)}, & \;\text{if}\;b_{ijk}>M_{ij}^{\prime } \end{array}\right),$$

where $M_{i}^{\prime }$ and $M_{ij}^{\prime }$ refer to the medians of *b_i _*and *b_ij_*, respectively.

### Sample selection

**Figure 2:**
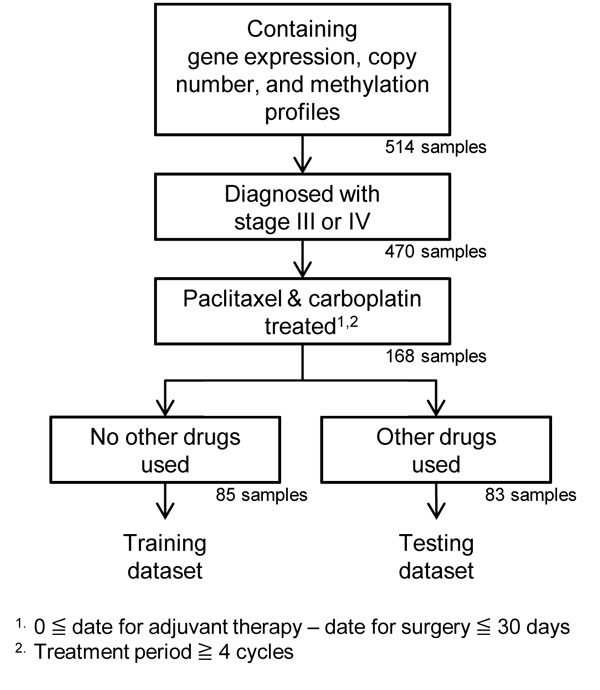
**The sample selection**. The rigorous process for sample selection.

After correcting the batch effect, a rigorous sample selection process was applied for finding the eligible samples for a targeted chemotherapy response. The ultimate goal of this step is to derive as many eligible samples as possible with the least possible confounding effects for both training and testing. To meet this requirement, clinical data were carefully examined. Since the criterion used for distinguishing a good response from a poor response was the progression free survival (PFS) time, which is defined in the next subsection, factors related to prognosis, such as tumor stages, drugs, and treatment time, were considered. As shown in Figure 2, we started with 514 samples profiled with all three techniques: gene expression, copy number, and methylation. Then, we considered samples only from advanced tumor stages (i.e., stage III and stage IV) and restricted samples to be quintessentially treated with paclitaxel and carboplatin: the treatment had to be started within 30 days after surgical resection and to last for at least 4 cycles. As a result, there were 168 samples which met this treatment requirement. A subset of 85 samples never treated with any other drugs before a failure event (tumor progression, tumor recurrence, death, or censored observation) were selected for training. Another subset of 83 samples treated with paclitaxel and carboplatin but involved in other treatment were selected for testing. The training/testing datasets were not chosen randomly since the 83 samples treated with other drugs may mislead the discovery of unique molecular patterns confounded by treatment effect. Nevertheless, these 83 samples are valuable for providing supporting evidence and justification if patterns or results found in training can also be observed in testing.

### Semi-supervised clustering

Semi-supervised clustering has been proposed and applied for identifying molecular subtypes in a variety of studies [16]. It outperforms unsupervised learning and naive splitting with an arbitrary survival threshold in detecting molecular subtypes since both the clinical outcome and the distribution overlapping between good prognosis and poor prognosis are considered [17]. Herein, a carefully designed semi-supervised clustering method is proposed. By using a log-rank survival test and a series of refinement processes, in which the gene expression data were embedded, significant features related to patients' survival were selected. With an additional scoring function for data discretization, classifications with similar patterns but distinct chemotherapy responses can be identified using classical hierarchical clustering. In this study, we focused on obtaining genetically altered genes, rather than transcriptionally dysregulated genes. However, we do require genes with copy number changes with concordant gene expression change, potentially driving the phenotypic change. Following this objective, the semi-supervised clustering was applied to both the copy number data and the methylation data, and the methods from feature selection to hierarchical clustering for both data sets were basically the same. We only detail the clustering steps for copy number data. The modifications for methylation data are demonstrated later.

#### Feature selection

To reduce suppression effects due to the genomic (or epigenomic) differences and maximize the ability of discriminating poor prognosis from good prognosis, a rigorous feature selection process was applied, in which, factors such as patient ages and tumor stages were carefully considered to minimize confounding effects.

A feature in copy number data refers to a gene in human genome, and the feature selection was done basically by a univariate log-rank test, which is a non-parametric hypothesis test comparing the survival functions of two groups of samples, and a series of feature refinements. First of all, we define PFS as the interval between the date of surgical resection and the date when a failure event occurred (i.e., disease progression). For samples without progression, recurrence, or death, the dates of last follow-up were considered as PFS with censoring. Second, based on the copy number ratio *c_ik _*for gene *i *from sample *k*, copy number statuses were categorized into gain (*c_ik _*> 0.4), normal (-0.5 ≤ *c_ik _*≤ 0.4), and loss (*c_ik _*< -0.5). Like a scanning process, the survival functions of the samples with normal copy number were tested against those with copy number gain (normal vs gain) as well as those with copy number loss (normal vs loss), feature by feature. To ensure the confidence in hypothesis testing, features leading to a small sample size (i.e., less than 15%, or less than 13) in either group were not considered, and features resulting in log-rank *p*-values less than 0.05 were then selected as candidates. After this, confounding factors such as ages and tumor stages were considered for feature refinement. Specifically, if the median of ages in both groups of samples were significantly different (*p*-values less than 0.05) in a Wilcoxon rank-sum detection test, or, if these two groups of samples' tumor stage assignment are distribute significantly differently (*p*-values less than 0.05 by Fisher's Exact test), then the selected features were removed from the candidates to eliminate possible detrimental effects from confounding factors. Moreover, the candidates were further refined using gene expression fold-changes. Similar to the scanning process used for survival comparison, the averaged gene expression levels derived from both groups of samples were compared, and the candidates causing expression fold-changes larger than 1.3 (or smaller than 1/1.3) were retained while those without significant expression fold-changes were disqualified for further consideration. We applied a relatively loose cutoff here only to preserve the concordance of gene expression alteration to the copy number change.

#### Data discretization

After feature selection, a scoring function was applied to the copy number data in the selected genes. Instead of using the original copy number ratios, which may be noisy and contain unrelated information, the hierarchical clustering scheme was applied to the data, discretized using a scoring function, called F-score, which transfers the grouping information and the survival conditions into discrete numbers, 1 (favorable), 0 (unknown), and -1 (unfavorable). Specifically, for a feature *i *selected from testing the gains against the normal, the F-score *F_ik _*for feature *i *and sample *k *on copy number data is given by

(5) $$F_{ik}=\{\begin{array}{ll} 1, & \;\text{if}\;c_{ik}\geq -0.5,\;k\in K_{Long}^{i}\\ \begin{array}{l} 0,\\ -1, \end{array} & \begin{array}{l} \;\text{if}\;c_{ik}<-0.5\;\text{or}\;c_{ik}=NaN\\ \;\text{if}\;c_{ik}\geq -0.5,\;k\in K_{Short}^{i} \end{array} \end{array},$$

where $K_{Long}^{i}$ and $K_{Short}^{i}$ refer to the samples with longer PFS survival and the samples with shorter PFS survival, respectively, determined by the log-rank test while evaluating the relation between PFS and CNAs for feature *i *(refer to Subsection Feature selection). Additionally, *c_ik _*= *NaN *refers to missing copy number ratios in feature *i *of sample *k*. Similarly, for a feature *i *selected from testing copy number losses against the normal, the F-score *F_ik _*for feature *i *and sample *k *on copy number data is given by

(6) $$F_{ik}=\{\begin{array}{ll} 1, & \;\text{if}\;c_{ik}\leq 0.4,\;k\in K_{Long}^{i}\\ 0, & \;\text{if}\;c_{ik}>0.4\;\text{or}\;c_{ik}=NaN\\ -1, & \;\text{if}\;c_{ik}\leq 0.4,\;k\in K_{Short}^{i} \end{array}.$$

The scores were assigned on a feature-by-feature basis. If a feature was selected twice in both cases (i.e., the gains versus the normal and the losses versus the normal), the F-scores were assigned according to the selection with smaller log-rank *p*-value.

#### Identification of molecular classifications

Based on the selected features and discretized copy number data, hierarchical clustering was applied for identifying the molecular classifications associated with chemotherapy response. The distances *d_kk' _*between samples *k *and *k' *were evaluated using the Jaccard coffiecient, which is given by

(7) $$d_{kk^{\prime }}=\frac{\#\left(F_{k}\neq F_{k^{\prime }}\right)}{\#\left[\left(F_{k}\neq 0\right)\vee \left(F_{k^{\prime }}\neq 0\right)\right]},$$

where *F_k _*indicates the vector of F-scores in sample *k*, *F_k' _*refers to the vector of F-scores in sample *k'*, and the length of these vectors are equal to the number of selected features. With all pairs of sample-sample distance being evaluated using Eq. (7), complete linkage was applied, and subsequent clusters derived. We used Kaplan-Meier analysis to check if these clusters exhibited significantly different survival distributions, and proposed molecular classifications if the log-rank *p*-value was less than 0.05.

#### Justification of selected features and identified classifications

The weighted *K*-Nearest Neighbor algorithm (weighted *K*NN) was applied to the testing datasets to justify the proposed classifications with the selected features. First of all, the testing samples were discretized in the F-scores using the same features and criterion mentioned in Subsection Data discretization. Then, the cluster of samples in the training datasets enriched with unfavorable scores were labeled as poor profiles (*l *= -1) while the other cluster of samples were labeled as good profiles (*l *= 1). Using the Jaccard coffiecients as sample-sample distances, the discriminant function *P_f _*for an independent sample *f *in the testing dataset was considered

(8) $$P_{f}=\frac{\overset{K}{\underset{h=1}{\sum }}\left(1/d_{fh}\right)l_{h}}{\overset{K}{\underset{h=1}{\sum }}1/d_{fh}},$$

where *l_h _*stands for the corresponding label of the sample *h*, and *d_fh _*denotes the distance between the sample *f *in the testing dataset and its *h*-th nearest sample in the training dataset, which is given by

(9) $$d_{fh}=\frac{\text{max}\left[\#\left(F_{f}\neq F_{h}\right),1\right]}{\#\left[\left(F_{f}\neq 0\right)\vee \left(F_{h}\neq 0\right)\right]},$$

where *F_f _*indicates the array of F-scores in sample *f *, and *F_h _*refers to the array of F-scores in sample *h*. The maximum function used in Eq. (9) is to ensure no zero in denominator in Eq. (8). The sample *f *in the testing dataset was classified as a good profile if *P_f _*> 0 or as a poor profile otherwise. In this study, *K *was chosen to be 3. Samples classified as good profiles as well as those samples classified as poor profiles were compared using the Kaplan-Meier survival analysis.

#### Modifications for methylation data

The proposed semi-supervised clustering was also applied to the methylation data. In order to fit the method, the batch-adjusted beta values for gene *i *from sample *k *were categorized into three statuses: hypermethylated $\left(b_{ik}^{*}>0.75\text{ quantile}\right)$, normal methylated $\left(0.25\leq b_{ik}^{*}\leq 0.75\text{ quantile}\right)$, and hypomethylated $\left(b_{ik}^{*}<0.25\text{ quantile}\right)$. Using the same criteria for log-rank testing, features (i.e., CpG sites) resulting in *p*-values less than 0.05 upon hypermethylated versus non-hypermethylated, or hypomethylated versus non-hypomethylated, were selected as candidates. Because of the continuous nature of beta values, the candidate refinement was moderately adjusted: if the beta values for a candidate feature exhibit a large correlation with the sample ages (Spearman correlation > 0.5), or if the beta values for the candidate feature derived from the stage III samples and the stage IV samples were significantly different in median by Wilcoxon rank-sum test, this candidate was removed from further consideration. Except for the aforementioned differences, other steps for identifying molecular classifications such as the candidate refinement using expression fold-change (larger than 1.3 or smaller than 1/1.3), F-scoring, and the hierarchical clustering (with the Jaccard coffiecient and complete linkage) remain the same. To illustrate the F-score $F_{ik}^{\prime }$ for feature *i *and sample *k *on methylation data more clearly, the following relation is provided. For a feature *i*, found PFS-related after feature refinement, the F-score $F_{ik}^{\prime }$ for sample *k *is given by

(10) $${F^{\prime }}_{ik}=\{\begin{array}{ll} 1, & \;\text{if}\;k\in K_{Long}^{i^{\prime }}\\ 0, & \;\text{if}\;b_{ik}^{*}=NaN\\ -1, & \;\text{if}\;k\in K_{Short}^{i^{\prime }} \end{array},$$

where $K_{Long}^{i}$ and $K_{Short}^{i}$ refer to the samples with longer survival and the samples with shorter survival, respectively, determined by the log-rank test while evaluating the relation between PFS and hypermethylation or hypomethylation in feature *i *in the feature selection for methylation data. Also, $b_{ik}^{*}=NaN$ refers to missing beta values in feature *i *of sample *k*. After data discretization, hierarchical clustering was applied to identify molecular classifications associated with chemotherapy response. Moreover, the testing datasets were also classified using the weighted *K*NN algorithm for classification justification.

### Identification of significant genes and ontologies

Once the molecular classifications associated with chemotherapy response were identified, differentially expressed genes in comparing the poor copy number profiles and the good profiles, or in comparing the poor methylation profiles and the good profiles, were detected using classical statistical approaches and methods for gene expression analysis. The good profiles mentioned herein study refer to the samples neither classified as poor copy number profiles nor classified as poor methylation profiles. Since confounding and suppression effects were reduced by the proposed procedure, more strict thresholds were simultaneously applied: fold change larger than 1.5 and *t*-test *p*-values less than 0.01. After deriving the significant gene set, enriched gene ontology terms (biological processes) were identified using the Gene Ontology Enrichment Analysis Software Toolkit (GOEAST) [18] available online at: http://omicslab.genetics.ac.cn/GOEAST/.

## Results

### Batch effect correction

**Figure 3:**
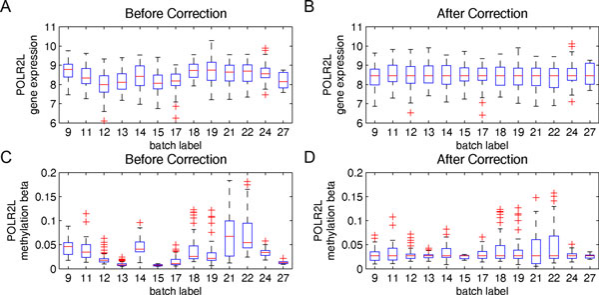
**The batch effect correction**. The box plot before and after the batch effect correction for the housekeeping gene POLR2L. (a) The original and (b) the adjusted gene expression values (in log_2_). (c) The original and (d) the adjusted methylation beta values.

To exemplify how the L/S adjustment modified the microarray data for batch effect correction, the gene expression values and methylation data of POLR2L, a gene encoding a subunit of RNA polymerase II, before and after the correction were compared in this section. POLR2L is one of the human housekeeping genes steadily expressed in cells. If the samples had been well assigned to random batches during the experimental design step, the medians and variances of the measures from different batches would approach the same values. To justify this, the PFS among all pairs of batches were compared and tested using the log-rank test; as a result, the PFS among most pairs of batches were found not significantly different (data is shown in the Additional file 1: Figure S1), indicating a random sample assignment of batches. However, as shown in Figures 3a and 3c, the measures from different batches, after normalization before batch effect correction, exhibit significant differences, particularly noticeable in medians (the red horizontal lines within the boxes). Figure 3b illustrates the gene expression data adjusted after batch effect correction using Eq. (1), and Figure 3d illustrates the methylation data adjusted after batch effect correction using Eq. (4). In these figures, the medians from different batches were aligned to the same values.

### Selected samples

From 514 tumor samples with gene expression, copy number, and methylation data, a subset of 85 samples were selected for training, and a subset of 83 samples were selected for testing according to the procedure outlined in Figure 2. As shown in the Additional file 1: Figure S2, the frequency of copy number alterations in the subset of 85 training samples is nearly the same as that in all 514 tumor samples, indicating no significant sampling bias in this regard due to the non-random selection.

The clinical characteristics of the 85 samples for training and the 83 samples for testing are generally identical except for the survival outcome. As shown in Table 1, the number of samples diagnosed in stage III and stage IV are 71 and 14 in the training dataset, respectively, while they are 68 and 15 in the testing dataset, respectively. Moreover, the minimum, maximum, and median age of the training dataset are 36, 87, and 58 years old, respectively, while these measures in the testing dataset are 34, 81, and 59 years old, respectively. However, as shown in Additional file 1: Figure S3, the testing samples show moderate improvement in PFS (log-rank *p*-value equal to 0.076), and the improvement might be attributed to the utilization of additional drugs other than paclitaxel and carboplatin. Considering treatments may affect PFS, especially with additional drugs that may prolong patients survival time, we set aside 83 samples only for testing purpose in order to avoid possible confounding effects, while maintaining 85 samples with paclitaxel and carboplatin treatment only to minimize the suppression effect at the same time during the training stage. By doing so, we optimized the trade-off between the sample size and analysis confidence, assuming that the 83 testing samples involving additional treatments preserve similar molecular signatures observed from the 85 training sample set.

**Table 1:** The characteristics of samples

Clinical characteristics	Measure	Training data	Testing data
Number of samples		85	83

Number of samples in stage III		71	68

Number of samples in stage IV		14	15

	min.	36	34
Ages (years old)	medium	58	59
	max.	87	81

	25th percentile	238	283.25
PFS (days)	median	396	458
	75th percentile	618.25	684

Number of samples with censored observation		14	22

Treatment		paclitaxel carboplatin	paclitaxel carboplatin other drugs

The clinical characteristics of the training samples and the testing samples.

### Molecular classifications identified using semi-supervised clustering

#### Selected features

**Figure 4:**
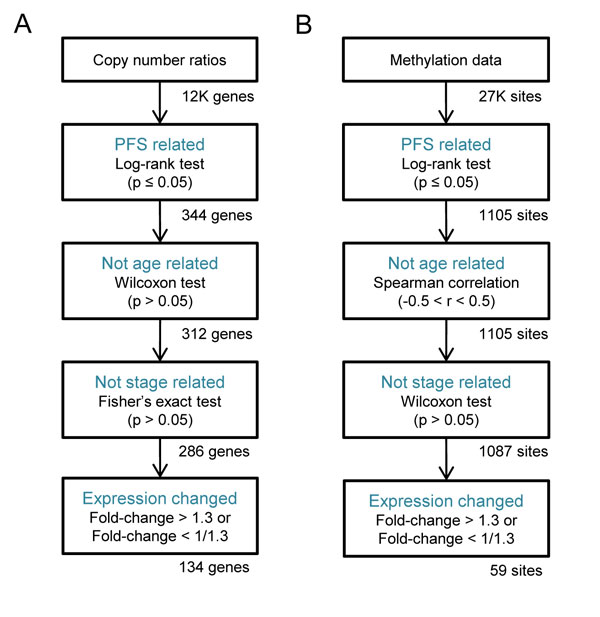
**The feature selection**. The filtering process of feature selection. The goal of feature selection is to detect features associated with PFS and gene expression changes but not related with patient ages and tumor stages. As a result, (a) 134 features (genes) were selected from copy number data, and (b) 59 features (CpG sites) were selected from methylation data.

As shown in Figure 4a, among 12,042 genes, 344 genes revealed significant differences in PFS with altered DNA copy numbers. After removing copy number changes that might be related to patient ages or tumor stages, there were 286 genes remained as candidates. Finally, gene expression concordance requirement (1.3 fold-change) reduced the set of candidate genes to 134. These 134 selected genes are listed in Additional file 1: Table S1. Similarly, 59 CpG sites were selected (the procedure is shown in Figure 4b) from the methylation data. The genes associated to the 59 CpG sites are listed in Additional file 1: Table S2.

#### Training results

**Figure 5:**
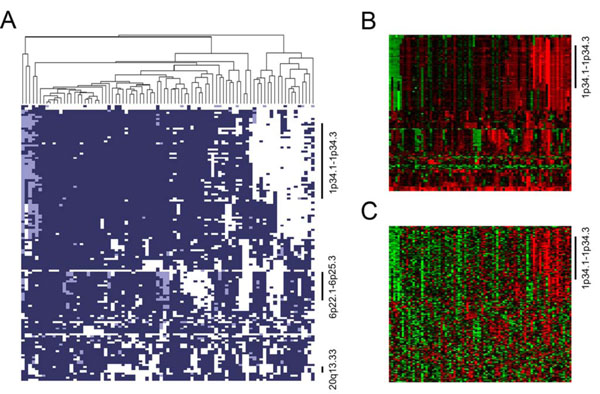
**The clustered results from the copy number profiles**. (a) The clustered results derived from the copy number profiles of the 85 training samples. Favorable scores (*F *= 1) are represented in dark blue; unfavorable scores (*F *= -1) are represented in white, and the unknown (*F *= 0) are represented in light blue. Columns are samples (merged by complete linkage), and rows are genes ordered by chromosomal positions. (b) The original copy number ratios in the same sample order and the same feature order. Positive ratios are shown in red, and negative ratios are shown in green, with the brightest red referring to copy number ratios larger than or equal to 2, the brightest green referring to a ratios smaller than or equal to -2, and the darkest color referring to a ratio of 0. (c) The z-transformed gene expression values in the same sample order and the same feature order. The red and green colors are shown in a same way but with different maximum and minimum, which are 1 and -1, respectively.

Figure 5a illustrates the clustered results upon copy number profiles using the proposed semi-supervised clustering method. In the figure, each column of the heatmap presents a copy number profile in 134 selected genes from one training sample. The columns are merged using the Jaccard distance derived from Eq. (7) with the complete linkage algorithm. The features displayed in rows are ordered by chromosomal positions from top to bottom. F-scores as 1 (favorable) derived from Eqs. (5) and (6) are shown in dark blue; F-scores as -1 (unfavorable) are shown in white, and F-scores as 0 are shown in light blue. The 85 training samples can be split into two distinct clusters: the right cluster with 18 samples enriched with unfavorable scores (termed 18 poor prognosis tumors, or 18 PPTs) and the left cluster with 67 samples enriched with favorable scores (termed 67 good prognosis tumors, or 67 GPTs). The majority of unfavorable scores in the right cluster corresponds to probes in chromosome 1p34.3 to 1p34.1, indicating a dominant region that might be associated with chemotherapy response. Figures 5b and 5c reveal the original copy number ratios and the z-transformed gene expression values, respectively, in the same order. As shown in Figure 5b, the enriched unfavorable scores mainly result from copy number amplifications ranging from 1p34.3 to 1p34.1. Also, up-regulation in gene expression corresponding to the copy number amplifications can be observed in Figure 5c.

**Figure 6:**
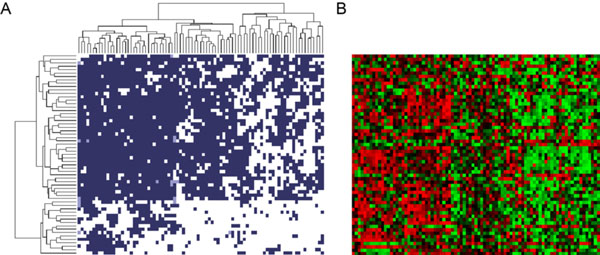
**The clustered results from the methylation profiles**. (a) The clustered results derived from the methylation profiles of the 85 training samples. Favorable scores (*F *= 1) are represented in dark blue; unfavorable scores (*F *= -1) are represented in white, and the unknown (*F *= 0) are represented in light blue. Columns are samples, and rows are CpG sites. Both columns and rows are merged by complete linkage. (b) The batch-adjusted methylation data in the same sample order and the same feature order. Among 85 beta values corresponding to a specific feature, the largest value (100th percentile) is shown in red, and the smallest (0th percentile) is shown in green. Also, the median (50th percentile) is shown in black, and all other values are revealed in color derived by linear-interpolation.

The clustered results on methylation profiles are shown in Figure 6a. Again, favorable scores are shown in dark blue; unfavorable scores are shown in white, and null values are shown in light blue. The columns are samples merged by Jaccard distance derived from Ed. (7) with complete linkage, similar to Figure 5a, and the rows stand for the 59 selected CpG sites significantly associated with PFS. Unlike rows in Figure 5a, rows in Figure 6a are ordered by hierarchical clustering. Similarly, the 85 training samples can be split into two clusters: one with 30 samples enriched with unfavorable scores, and one with 55 samples enriched with favorable scores (30 PPTs and 55 GPTs, respectively). Figure 6b shows the batch-adjusted methylation data. For every 85 measures in a row, the smallest value (0th percentile) is shown in green, and the largest value (100th percentile) is shown in red. In addition, the median (50th percentile) is shown in black, and other values are shown in colors derived from linear interpolation. As shown in the figure, the selected CpG sites are generally hypomethylated in the 30-sample cluster. However, for those genes corresponding to the selected CpG sites, no significant difference in gene expression is observed.

**Figure 7:**
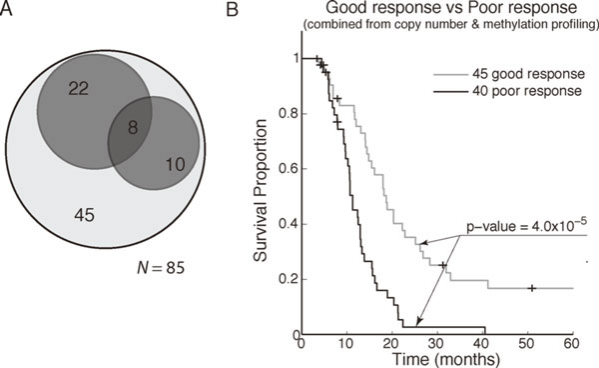
**The training results**. (a) The Venn diagram demonstrates the clustered results derived from the 85 training samples. Among the 85 training samples, a total of 40 samples, 18 with poor copy number profiles and 30 with poor methylation profiles, were detected as PPTs; the other 45 samples without either poor copy number profiles or poor methylation profiles were grouped as GPTs. (b) The Kaplan-Meier curves reveal distinct PFS functions between the union of the 40 poor profiles and the 45 good profiles.

The training results derived from both the copy number data and the methylation data are integrated and shown in Figure 7. A Venn diagram, shown in Figure 7a, illustrates that 18 PPTs (in the right dark gray circle) demarcated by the amplifications in 1p34.3 - 1p34.1 and 30 PPTs (in the above dark gray circle) due to hypomethylation in specific CpG sites, with only 8 samples overlapping, result in total of 40 PPTs. Other than these PPTs, 45 GPTs determined by both copy number and methylation profiles are shown in light gray. The 40 PPTs reveal distinct difference in PFS while comparing with 45 GPTs. As shown in Figure 7b, where the survival function of 40 PPTs is shown in black and the survival function of 45 GPTs is shown in light gray, the difference in PFS between the black and the light gray is significant with log-rank *p*-value of 4.0 × 10^-5^, indicating the potential existence of molecular subtypes with distinct chemotherapy response.

#### Testing results

**Figure 8:**
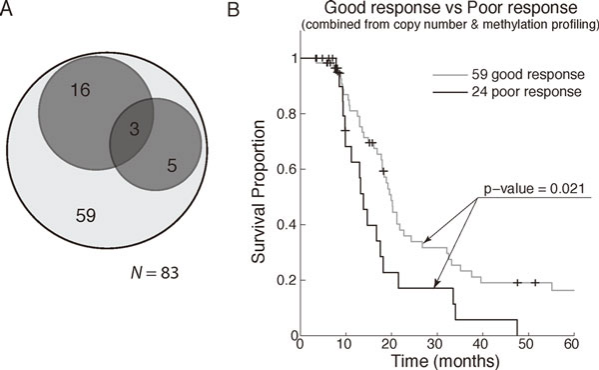
**The testing results**. (a) The Venn diagram demonstrating the clustered results derived from the 83 testing samples. Among the 83 training samples, a total of 24 samples, 8 with poor copy number profiles and 19 with poor methylation profiles, were classified as PPTs; the other 59 samples without either poor copy number profiles or poor methylation profiles were classified as GPTs. (b) The Kaplan-Meier curves reveal distinct PFS functions between the union of the 24 poor profiles and the 59 good profiles.

To justify the selected features and identified molecular classifications derived from the training samples, the weighted *K*NN algorithm detailed previously was applied to 83 independent testing samples, and the classified results based on both the copy number profiles and the methylation profiles were integrated and shown in Figure 8a. As shown in the Venn diagram, 8 PPTs classified using copy number profiles (in the right dark gray circle) and 19 PPTs classified using methylation profiles (in the above dark gray circle), with a small extent of overlapping (3 samples), result in a total of 24 PPTs. With the remaining 59 GPTs either classified using copy number profiles or methylation profiles, the PFS of the poor and the good prognosis were compared and tested. As shown in Figure 8b, the log-rank *p*-value comparing the light gray (good) to the black (poor) is 0.021, and the Kaplan-Meier curves in testing reveal similar patterns to those in the training. These results validate the finding we derived from the training dataset.

### Significant genes and enriched ontologies

Significant genes and enriched ontologies were identified using classical statistical approaches and an enrichment analysis. Based on the training results, as shown in the Venn Diagram and the Kaplan-Meier curves in Figure 7, two molecular classifications associated with poor chemotherapy response (18 PPTs from copy number data and 30 PPTs from methylation data) and one group of samples with good chemotherapy response were identified. We begin with a comparison of the 18 PPTs derived from copy number profiles to the 45 GPTs. Using *t*-test and expression fold-change, 107 differentially expressed genes between these two groups of samples were detected, and the top 15 differentially expressed genes ranked by *t*-test *p*-values are shown in Additional file 1: Table S3. Moreover, significant biological processes were subsequently identified using GOEAST [18]. The top 15 biological processes ranked by *p*-values are listed in Additional file 1: Table S4, where all of the listed biological processes resulted in a *p*-value less than 1.8 × 10^-5^. The other 30 PPTs derived from methylation profiles were also compared to the 45 GPTs, and 34 differentially expressed genes were detected using *t*-test and expression fold-change. Similarly, the top 15 differentially expressed genes ranked by *t*-test *p*-values are shown in Additional file 1: Table S5, and the top 15 significant altered biological processes are listed in Additional file 1: Table S6.

## Discussion and conclusions

As high-throughput technologies such as microarray and short-read sequencing become more and more popular in biological studies, the complexity and dimensions these datasets possess make the statistical analysis more difficult. Additionally, extraneous variables resulting from sample variability may hinder analysts from avoiding false discovery and subsequently achieving reliable results. Dealing with these extraneous variables, whether confounding or suppression, is challenging. To address these issues, we proposed a procedure for the reduction of confounding and suppression effects, in which a batch effect correction, a sample selection process, and a semi-supervised clustering method were considered. The batch effect correction used an L/S adjustment to eliminate confounding effects due to experimental differences, and the sample selection was designed to focus on single chemotherapy, reducing variability due to different treatments. After batch effect correction and sample selection, a novel semi-supervised clustering method that further addressed the confounding from ages and tumor stages was applied, and gene expression values, DNA copy number ratios, methylation data, and clinical information were analyzed for unraveling molecular classifications associated with chemotherapeutic response. As a result, significant genes and enriched ontologies were identified.

The batch effect due to experimental processing is a known issue in microarray studies, and there has been several attempts to correct the batch effect for gene expression profiling experiments. For example, the software "COMBAT" [14] utilized empirical Bayes frameworks for adjusting the batch effect in gene expression particularly when the sample size is small. Despite the existence of sophisticated algorithms for batch adjustment in gene expression, we applied the simple L/S method for batch effect correction, since the TCGA dataset has large sample size (larger than 25) in most batches. Moreover, the simple L/S method can be easily extended and applied to the methylation beta values without causing much difficulty and computational burdens. To further improve the L/S method, one could consider to apply the median of absolute deviation (MAD) instead of Eq. (2) for estimating the standard deviation in a more robust way [19].

The molecular classifications were identified from copy number and methylation profiles. CNAs are common genomic aberrations shown to associate with mRNA expression level changes, gene function modifications, and significant differences in prognosis of a variety of tumors [20]. Also, methylation status has been implicated in affecting the drug response [21]. Since these genomic or epigenomic mutations do not necessarily lead to linear changes in gene expression levels [22,23], expression fold-change rather than Pearson correlation was applied to evaluate their effects on transcription. For the semi-supervised clustering, the log-rank test was initially applied to select significant features associated with PFS. After removing features not affecting transcription or potentially correlated with ages and stages, a scoring function was applied to discretize and denoise data. Then, the classical hierarchical clustering method was applied, and two molecular classifications associated with poor chemotherapy response were identified. Interestingly, in the classification detected from copy number profiles, unfavorable scores were enriched in the region of 1p34.3 - 1p34.1, indicating a dominant CNA segment that might affect chemotherapy response. Also, in the classifi-cation detected from methylation profiles, samples were generally hypomethylated in the selected CpG sites. It is a good opportunity to further examine these regions for a better understanding of chemoresistance.

Differentially expressed genes detected by comparing the tumors with poor prognosis to the samples with good prognosis provide us candidate genes for studying the functions or mechanisms differentiating chemotherapy response. Comparing with the 14 genes selected by Hartmann *et al. *[10] for predicting the platinum-paclitaxel chemotherapy response, we found SF3A3 was also detected in this study. Moreover, many candidate genes such as GNL2, RRAGC, RFC3, and ENC1 listed in Additional file 1: Table S3 and Table S5 have also been reported as being differentially expressed in a related *in vitro *study [24]. Obviously, these genes require further validation in order to confirm their chemotherapy response. In addition, genes involved in associated biological processes, such as microtubule polymerization or depolymerization, urogenital system development, were detected using GOEAST. These results could be re-assessed with network modeling, for example, the Boolean Network (BN) and the Probabilistic Boolean Networks (PBN). Further mathematical modeling of this type is an essential step to uncover the underlying mechanism of chemoresistance in these tumors, to provide better prognosis, and ultimately to improve the care of ovarian cancer patients.

The proposed procedure can help reduce confounding for mining useful information; it is powerful but not omnipotent. In fact, the best way to achieve a confident conclusion may rely on rigorous experimental designs, not simply relying on state-of-the-art statistical analysis techniques. In other words, the less confounding factors an experiment has, the more reliable results an experimentalist can generally achieve through a case-control study.

## Competing interests

The authors declare that they have no competing interests.

## Authors' contributions

FH, TH, and YC designed the methods for data analysis. ES, AB and ED participated in the formulation of research topic. All authors collectively wrote the paper, read and approved the final manuscript.

## Supplementary Material

Additional file 1 **Supplementary figures and tables**. This additional file contains supplementary figures and tables mentioned in the study, including Figures S1-S3 and Tables S1-S6. Click here for file

## References

[B1] Huang J Zhang L Frequent genetic abnormalities of the PI3K/AKT pathway in primary ovarian cancer predict patient outcome Genes Chromosomes Cancer 2011 50 606 618 10.1002/gcc.20883 21563232 PMC3110626

[B2] Bowtell D The genesis and evolution of high-grade serous ovarian cancer Nature Reviews Cancer 2010 10 11 803 808 10.1038/nrc2946 20944665

[B3] Bookman M Standard treatment in advanced ovarian cancer in 2005: the state of the art International Journal of Gynecological Cancer 2005 15 212 220 10.1111/j.1525-1438.2005.00444.x 16343233

[B4] Joerger M Huitema A Population pharmacokinetics and pharmacodynamics of paclitaxel and carboplatin in ovarian cancer patients: a study by the European organization for research and treatment of cancer-pharmacology and molecular mechanisms group and new drug development group Clinical Cancer Research 2007 13 21 6410 10.1158/1078-0432.CCR-07-0064 17975154

[B5] Vella N Aiello M 'Genetic profiling'and ovarian cancer therapy (Review) Molecular medicine reports 2011 4 771 777 21687948 10.3892/mmr.2011.512

[B6] Xiao H Verdier-Pinard P Insights into the mechanism of microtubule stabilization by Taxol Proc Natl Acad Sci U S A 2006 103 27 10166 10173 10.1073/pnas.0603704103 16801540 PMC1502429

[B7] Wang D Lippard S Cellular processing of platinum anticancer drugs Nature Reviews Drug Discovery 2005 4 4 307 320 10.1038/nrd1691 15789122

[B8] Fung-Kee-Fung M Oliver T Elit L Oza A Hirte H Bryson P Optimal chemotherapy treatment for women with recurrent ovarian cancer Current Oncology 2007 14 5 195 10.3747/co.2007.148 17938703 PMC2002482

[B9] Jazaeri A Awtrey C Gene expression profiles associated with response to chemotherapy in epithelial ovarian cancers Clinical cancer research 2005 11 17 6300 10.1158/1078-0432.CCR-04-2682 16144934

[B10] Hartmann L Lu K Gene expression profiles predict early relapse in ovarian cancer after platinum-paclitaxel chemotherapy Clinical cancer research 2005 11 6 2149 10.1158/1078-0432.CCR-04-1673 15788660

[B11] Etemadmoghadam D deFazio A Integrated genome-wide DNA copy number and expression analysis identifies distinct mechanisms of primary chemoresistance in ovarian carcinomas Clinical Cancer Research 2009 15 4 1417 10.1158/1078-0432.CCR-08-1564 19193619 PMC2670486

[B12] Network TCGA Integrated genomic analyses of ovarian carcinoma Nature 2011 474 609 10.1038/nature10166 21720365 PMC3163504

[B13] Leek J Scharpf R Bravo H Simcha D Langmead B Johnson W Geman D Baggerly K Irizarry R Tackling the widespread and critical impact of batch effects in high-throughput data Nature Reviews Genetics 2010 11 10 733 739 10.1038/nrg2825 PMC3880143 20838408

[B14] Li C Rabinovic A Adjusting batch effects in microarray expression data using empirical Bayes methods Biostatistics 2007 8 118 127 10.1093/biostatistics/kxj037 16632515

[B15] Du P Zhang X Huang C Jafari N Kibbe W Hou L Lin S Comparison of Beta-value and M-value methods for quantifying methylation levels by microarray analysis BMC bioinformatics 2010 11 587 10.1186/1471-2105-11-587 21118553 PMC3012676

[B16] Koestler D Marsit C Christensen B Karagas M Bueno R Sugarbaker D Kelsey K Houseman E Semi-supervised recursively partitioned mixture models for identifying cancer subtypes Bioinformatics 2010 26 20 2578 10.1093/bioinformatics/btq470 20834038 PMC2951086

[B17] Bair E Tibshirani R Semi-supervised methods to predict patient survival from gene expression data PLoS biology 2004 2 4 e108 10.1371/journal.pbio.0020108 15094809 PMC387275

[B18] Zheng Q Wang X GOEAST: a web-based software toolkit for Gene Ontology enrichment analysis Nucleic acids research 2008 36 suppl 2 W358 18487275 10.1093/nar/gkn276 PMC2447756

[B19] Hsu F Chen H Tsai M Lai L Huang C Tu S Chuang E Chen Y A model-based circular binary segmentation algorithm for the analysis of array CGH data BMC Research Notes 2011 4 394 10.1186/1756-0500-4-394 21985277 PMC3224564

[B20] Pinkel D Albertson D Array comparative genomic hybridization and its applications in cancer Nature genetics 2005 37 S11 S17 10.1038/ng1569 15920524

[B21] Li M Balch C Montgomery J Jeong M Chung J Yan P Huang T Kim S Nephew K Integrated analysis of DNA methylation and gene expression reveals specific signaling pathways associated with platinum resistance in ovarian cancer BMC medical genomics 2009 2 34 10.1186/1755-8794-2-34 19505326 PMC2712480

[B22] Hsu FH Serpedin E Chen Y Dougherty ER Stochastic modeling of the relationship between copy number and gene expression based on transcriptional logic IEEE Transactions on Biomedical Engineering 2012 59 272 280 22042124 10.1109/TBME.2011.2173341

[B23] Houshdaran S Hawley S Palmer C Campan M Olsen M Ventura A Knudsen B Drescher C Urban N Brown P DNA methylation profiles of ovarian epithelial carcinoma tumors and cell lines PloS one 2010 5 2 e9359 10.1371/journal.pone.0009359 20179752 PMC2825254

[B24] Konstantinopoulos P Fountzilas E Pillay K Zerbini L Libermann T Cannistra S Spentzos D Carboplatin-induced gene expression changes in vitro are prognostic of survival in epithelial ovarian cancer BMC medical genomics 2008 1 59 10.1186/1755-8794-1-59 19038057 PMC2613398

[B25] Hsu F Serpedin E Hsiao T Bishop AJR Dougherty ER Chen Y Identifying genes associated with chemotherapy response in ovarian carcinomas based on DNA copy number and expression profiles Genomic Signal Processing and Statistics (GENSIPS), 2011 IEEE International Workshop on: 4-6 December 2011 2011 46 49 10.1109/GENSiPS.2011.6169438

