# Supplementary material for: Reducing confounding and suppression effects in TCGA data: an integrated analysis of chemotherapy response in ovarian cancer
Source: BMC Genomics. 2012 Oct 26;13(Suppl 6):S13. doi: 10.1186/1471-2164-13-S6-S13 (PMC3481440; doi:10.1186/1471-2164-13-S6-S13)
Supplement: Additional file 1 — Supplementary figures and tables. This additional file contains supplementary figures and tables mentioned in the study, including Figures S1-S3 and Tables S1-S6. [file 1471-2164-13-S6-S13-S1.pdf]

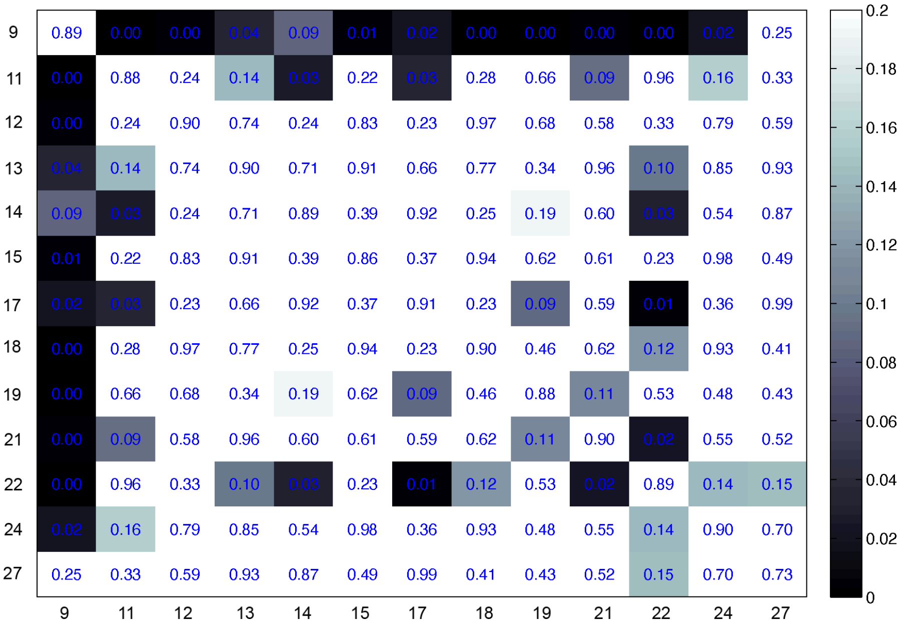

Figure S1. The log-rank p-values testing the PFS in all pairs of batches. Except for batch no.9, which contains significant poor PFS, most pairs of batches do not have significant differences in PFS.

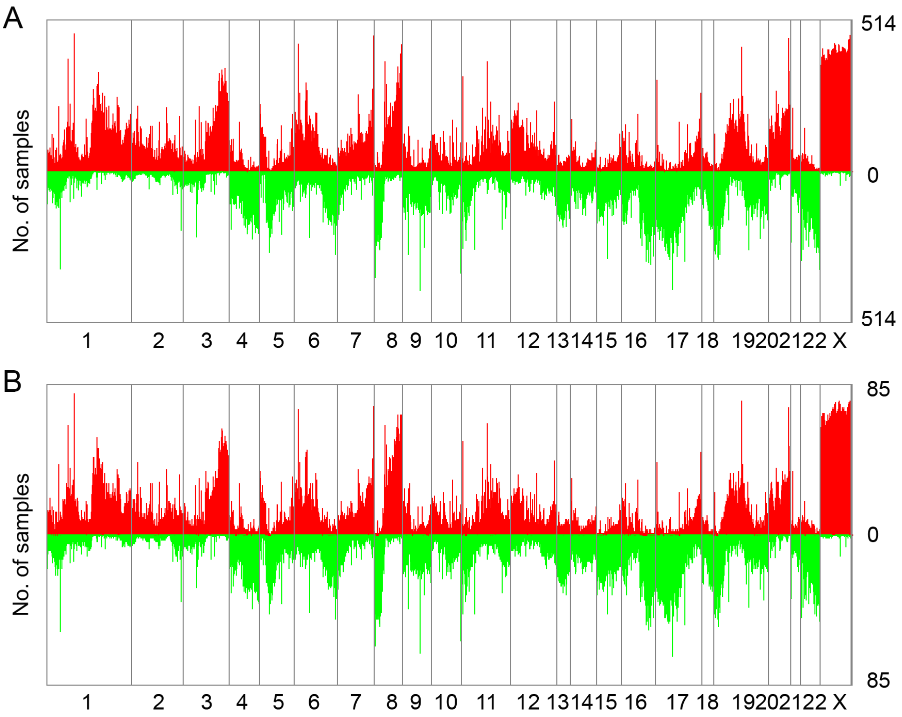

Figure S2. The frequency of copy number gains (red) and copy number losses (green) in (a) 514 TCGA ovarian tumor samples, and (b) the subset of 85 training samples.

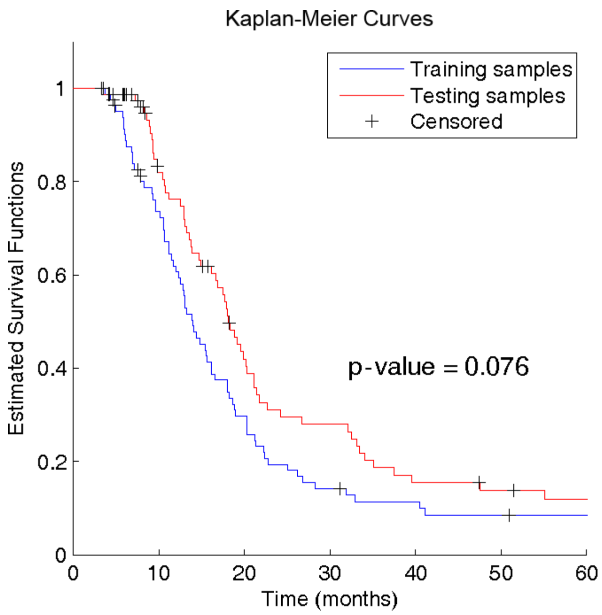

Figure S3. The PFS functions of the 85 training samples and the 83 testing samples.

Table S1. 134 features (genes) selected from the copy number profiles of the 85 training samples.

Gene symbol	Cytoband	Gene symbol	Cytoband	Gene symbol	Cytoband
MAP3K6	1p36.11	UROD	1p34.1	E2F3	6p22.3
FGR	1p36.11	MUTYH	1p34.1	C6orf62	6p22.3
KPNA6	1p35.1	TESK2	1p34.1	HMGN4	6p22.2
EIF3I	1p35.1	FAF1	1p32.3	ZSCAN16	6p22.1
ZMYM6	1p34.3	RNF11	1p32.3	PPP1R11	6p22.1
ZMYM1	1p34.3	EPS15	1p32.3	TAPBP	6p21.32
ZMYM4	1p34.3	OSBPL9	1p32.3	FANCE	6p21.31
KIAA0319L	1p34.3	NRD1	1p32.3	RNF8	6p21.2
PSMB2	1p34.3	ECHDC2	1p32.3	C6orf64	6p21.1
EIF2C1	1p34.3	CPT2	1p32.3	PPP2R5D	6p21.1
EIF2C3	1p34.3	MAGOH	1p32.3	ZNF394	7q22.1
TRAPPC3	1p34.3	DIO1	1p32.3	TAF6	7q22.1
MAP7D1	1p34.3	C1orf41	1p32.3	PCOLCE	7q22.1
MRPS15	1p34.3	LRRC42	1p32.3	WDR60	7q36.3
C1orf149	1p34.3	TMEM59	1p32.3	PDLIM2	8p21.3
SNIP1	1p34.3	SSBP3	1p32.3	C8orf33	8q24.3
GNL2	1p34.3	USP24	1p32.3	RPS24	10q22.3
C1orf109	1p34.3	USP1	1p31.3	MANSC1	12p13.2
YRDC	1p34.3	ITGB3BP	1p31.3	KCNJ8	12p12.1
MTF1	1p34.3	GNG12	1p31.3	C12orf35	12p11.21
SF3A3	1p34.3	ISG20L2	1q23.1	BICD1	12p11.21
RRAGC	1p34.3	ALDH9A1	1q24.1	PDIA3	15q15.3
MYCBP	1p34.3	KIFAP3	1q24.2	MT1G	16q13
C1orf108	1p34.3	CHI3L1	1q32.1	PSMC5	17q23.3
MACF1	1p34.3	PRELP	1q32.1	C18orf10	18q12.2
PABPC4	1p34.2	NUCKS1	1q32.1	ZNF430	19p12
PPIE	1p34.2	MIA3	1q41	FXYD3	19q13.12
TRIT1	1p34.2	RBM34	1q42.3	USF2	19q13.12
CAP1	1p34.2	DNAJC10	2q32.1	C20orf29	20p13
PPT1	1p34.2	C2orf47	2q33.1	PLCB4	20p12.3
RLF	1p34.2	PIK3R4	3q22.1	ANKRD5	20p12.2
ZMPSTE24	1p34.2	ATP2C1	3q22.1	C20orf4	20q11.23
NFYC	1p34.2	ASTE1	3q22.1	TGIF2	20q11.23
CTPS	1p34.2	DBR1	3q22.3	C20orf24	20q11.23
SCMH1	1p34.2	CEP70	3q22.3	KIAA1219	20q11.23
FOXJ3	1p34.2	NOLA2	5q35.3	ACTR5	20q11.23
C1orf50	1p34.2	EXOC2	6p25.3	SLMO2	20q13.32
SLC2A1	1p34.2	GMDS	6p25.3	LSM14B	20q13.33
EBNA1BP2	1p34.2	NQO2	6p25.2	PSMA7	20q13.33
CDC20	1p34.2	DSP	6p24.3	OSBPL2	20q13.33
KIAA0467	1p34.2	TFAP2A	6p24.3	STS	Xp22.31
IPO13	1p34.1	HIVEP1	6p24.1	BEX4	Xq22.1
C1orf164	1p34.1	NOL7	6p23	MORC4	Xq22.3
TMEM53	1p34.1	RANBP9	6p23	MOSPD1	Xq26.3
EIF2B3	1p34.1	MYLIP	6p22.3		

Table S2. 59 features (CpG sites) selected from the methylation profiles of the 85 training samples.

Gene symbol	Chromosome	Position	Gene symbol	Chromosome	Position
CYP4B1	1	47037188	GPR126	6	142665126
ADORA3	1	111848438	THBS2	6	169395886
NRAS	1	115061191	CPVL	7	29152635
MLLT11	1	149297592	PDK4	7	95063456
S100A6	1	151775484	ZNF282	7	148523595
S100A1	1	151867489	ACTR3B	7	152087633
NES	1	154913277	CA9	9	35663909
CCDC19	1	158136584	COL5A1	9	136672962
CD48	1	158948384	CALML5	10	5532328
FMOD	1	201587009	C10orf116	10	88718258
STEAP3	2	119698430	ABLIM1	10	116434030
FAP	2	162808285	IGF2	11	2110567
CYBRD1	2	172086932	IGF2	11	2118049
COL3A1	2	189547719	LDHC	11	18390591
CXCR7	2	237143403	BBOX1	11	27018992
CHL1	3	213161	MS4A6A	11	59706981
PDZRN3	3	73756760	CTSF	11	66092225
ALCAM	3	106568681	ALDH3B1	11	67534528
PLOD2	3	147362332	CRYAB	11	111287226
STX18	4	4594917	LCP1	13	45655396
MSX1	4	4912231	SUCLA2	13	47473401
RHOH	4	39874990	HCFC1R1	16	3014171
IL8	4	74825329	PYCARD	16	31121918
CFI	4	110942151	CCL5	17	31231445
PGRMC2	4	129428175	TOP2A	17	35828092
PAM	5	102229444	MOCOS	18	32021224
TGFBI	5	135392451	SERPINB5	18	59308458
CYFIP2	5	156629098	ADAMTS5	21	27261313
CFB	6	32022284	HMGN1	21	39642786
GCLC	6	53518141			

Table S3. Top 15 differentially expressed genes (by t-test p-value) derived by comparing the 18 PPTs to the 45 GPTs using the criteria: t-test p-value less than 0.01 and expression fold-change larger than 1.5.

Gene symbol	Expression fold-change ($\log_2$)	p-value
C1orf149	1.07	8.3×10^{-11}
PPIE	0.86	1.3×10^{-10}
GNL2	1.10	1.2×10^{-9}
RRAGC	1.04	1.8×10^{-9}
C1orf109	1.15	2.6×10^{-9}
TRIT1	0.69	3.3×10^{-9}
PABPC4	1.10	6.0×10^{-9}
UTP11L	0.89	1.4×10^{-8}
SNIP1	0.68	1.8×10^{-8}
NDUFS5	0.84	2.1×10^{-8}
DPH2	0.62	2.2×10^{-8}
PPCS	0.77	2.4×10^{-8}
JMJD2A	0.78	2.7×10^{-8}
MYCBP	1.08	2.7×10^{-8}
YBX1	0.76	3.9×10^{-8}

Table S4. Top 15 enriched biological processes (by p-value) detected by GOEAST using 107 differentially expressed genes derived by comparing the 18 PPTs to the 45 GPTs with the criteria: t-test p-value less than 0.01 and expression fold-change larger than 1.5. Note: log OR refers to log odds ratio.

GOID	Term	Log OR	p-value
GO:0002051	osteoblast fate commitment	6.95	2.2×10^{-8}
GO:0001649	osteoblast differentiation	4.26	9.2×10^{-8}
GO:0050775	positive regulation of dendrite morphogenesis	5.59	2.5×10^{-7}
GO:0017182	peptidyl-diphthamide metabolic process	5.95	5.5×10^{-7}
GO:0017183	peptidyl-diphthamide biosynthetic process from peptidyl-histidine	5.95	5.5×10^{-7}
GO:0061036	positive regulation of cartilage development	5.87	7.8×10^{-7}
GO:0009653	anatomical structure morphogenesis	1.48	2.3×10^{-6}
GO:0001710	mesodermal cell fate commitment	5.56	2.7×10^{-6}
GO:0001667	ameboidal cell migration	3.97	6.7×10^{-6}
GO:0018202	peptidyl-histidine modification	5.31	6.7×10^{-6}
GO:0048333	mesodermal cell differentiation	5.31	6.7×10^{-6}
GO:0097070	ductus arteriosus closure	6.78	1.6×10^{-5}
GO:0001503	ossification	3.11	1.6×10^{-5}
GO:0001655	urogenital system development	2.79	1.7×10^{-5}
GO:0006898	receptor-mediated endocytosis	3.76	1.8×10^{-5}

Table S5. Top 15 differentially expressed genes (by t-test p-value) derived by comparing the 30 PPTs to the 45 GPTs using the criteria: t-test p-value less than 0.01 and expression fold-change larger than 1.5.

Gene symbol	Expression fold-change ($\log_2$)	p-value
ALG5	-0.61	7.8×10^{-5}
GCHFR	-0.67	1.5×10^{-4}
SOBP	-0.76	2.8×10^{-4}
CXCR7	-1.17	3.3×10^{-4}
GLRX	-0.61	4.1×10^{-4}
RFC3	-0.63	6.5×10^{-4}
FKBP11	-0.78	9.3×10^{-4}
KDELC1	-0.62	1.1×10^{-3}
EPHB3	-0.70	1.2×10^{-3}
TM9SF2	-0.60	1.5×10^{-3}
ENC1	-0.70	1.7×10^{-3}
HPN	0.72	1.8×10^{-3}
LY96	-0.78	2.0×10^{-3}
NPR1	0.60	2.4×10^{-3}
ITM2C	-0.98	2.5×10^{-3}

Table S6. Top 15 enriched biological processes (by p-value) detected by GOEAST using 34 differentially expressed genes derived by comparing the 30 PPTs to the 45 GPTs with the criteria: t-test p-value less than 0.01 and expression fold-change larger 1.5. Note: log OR refers to log odds ratio.

GOID	Term	Log OR	p-value
GO:0021957	corticospinal tract morphogenesis	8.83	5.3×10^{-10}
GO:0007413	axonal fasciculation	7.22	3.7×10^{-9}
GO:0032314	regulation of Rac GTPase activity	7.28	3.7×10^{-9}
GO:0048681	negative regulation of axon regeneration	8.24	3.7×10^{-9}
GO:0060561	apoptosis involved in morphogenesis	7.95	8.1×10^{-9}
GO:0032317	regulation of Rap GTPase activity	7.95	8.1×10^{-9}
GO:0032487	regulation of Rap protein signal transduction	7.95	8.1×10^{-9}
GO:0070571	negative regulation of neuron projection regeneration	7.95	8.1×10^{-9}
GO:2000378	negative regulation of reactive oxygen species metabolic process	7.83	1.2×10^{-8}
GO:0021952	central nervous system projection neuron axonogenesis	6.82	1.2×10^{-8}
GO:0035020	regulation of Rac protein signal transduction	6.68	1.9×10^{-8}
GO:0007021	tubulin complex assembly	7.61	2.2×10^{-8}
GO:0031109	microtubule polymerization or depolymerization	7.61	2.2×10^{-8}
GO:0048731	system development	1.90	2.8×10^{-8}
GO:0008038	neuron recognition	6.47	3.8×10^{-8}
